# Supplementary material for: Integration of epigenetic and genetic profiles identifies multiple sclerosis disease-critical cell types and genes
Source: Commun Biol. 2023 Mar 30;6:342. doi: 10.1038/s42003-023-04713-5 (PMC10063586; doi:10.1038/s42003-023-04713-5)
Supplement: Supplementary file 2 — Supplementary Information [file 42003_2023_4713_MOESM2_ESM.pdf]

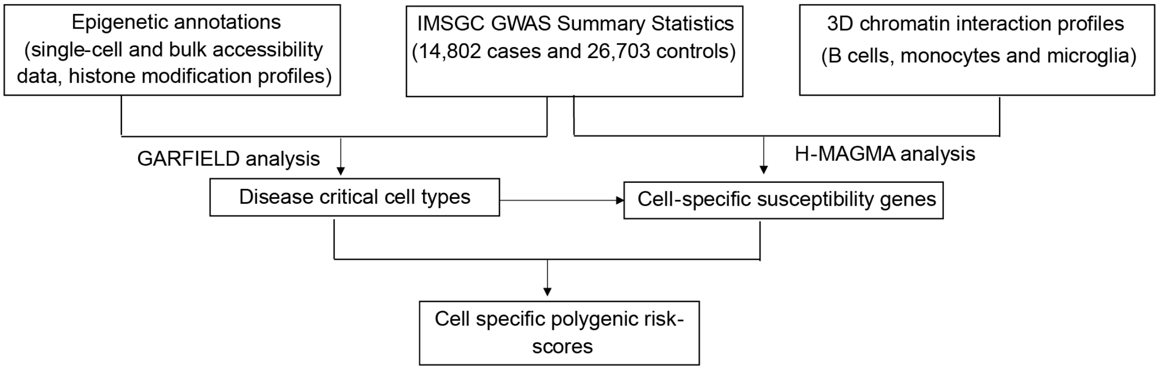

Supplementary Fig. 1. Schematic representation the overview of bioinformatic analysis.

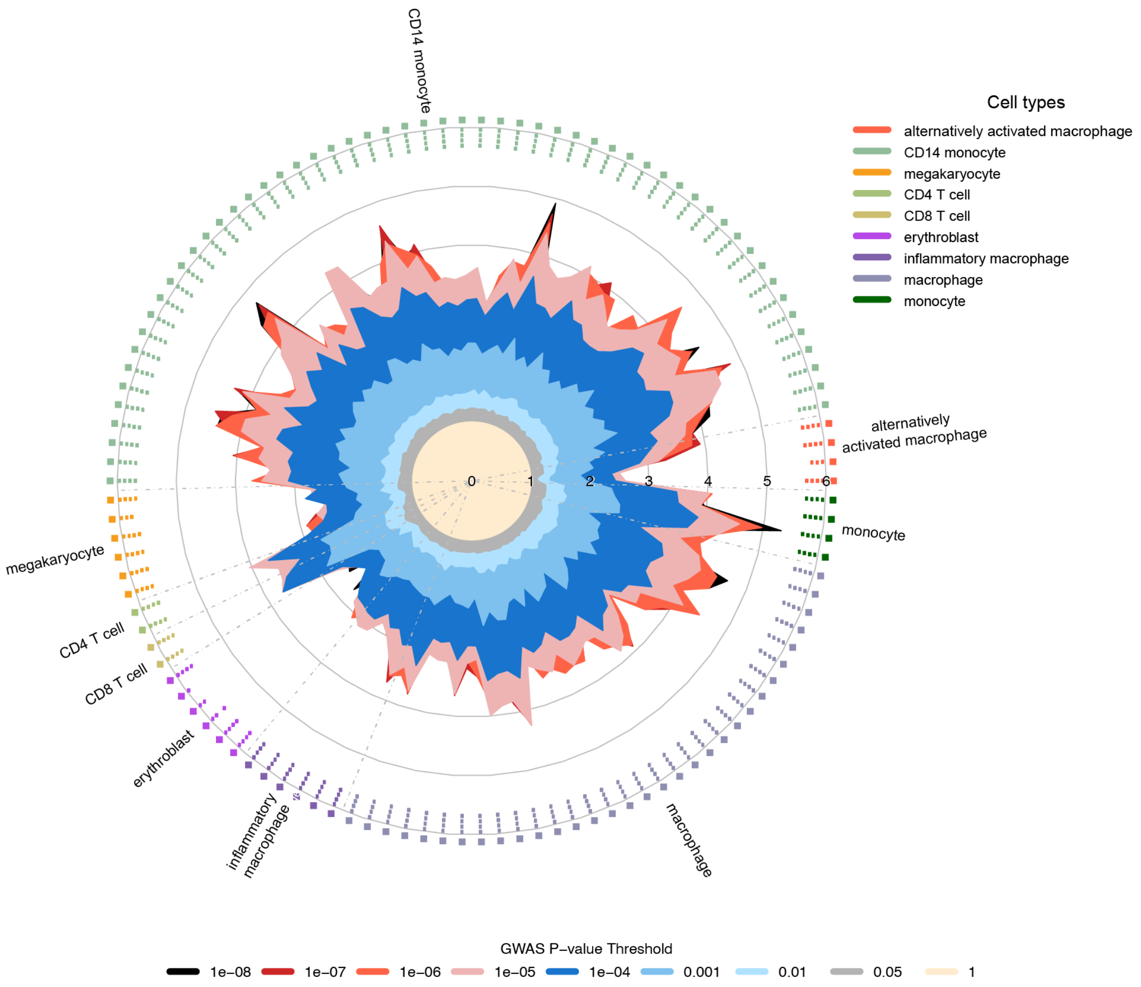

Supplementary Fig. 2. Enrichment of MS GWAS associations in open chromatin regions (OCRs). Radial lines show odds ratio (OR) values at eight GWAS P-value thresholds (T) for immune cell types available from the Blueprint project. Dots in the inner ring of the outer circle denote significant GARFIELD enrichment (if present) at T < 10−5 (outermost) to T < 10−8 (innermost) after multiple-testing correction. The colors represent cell types.

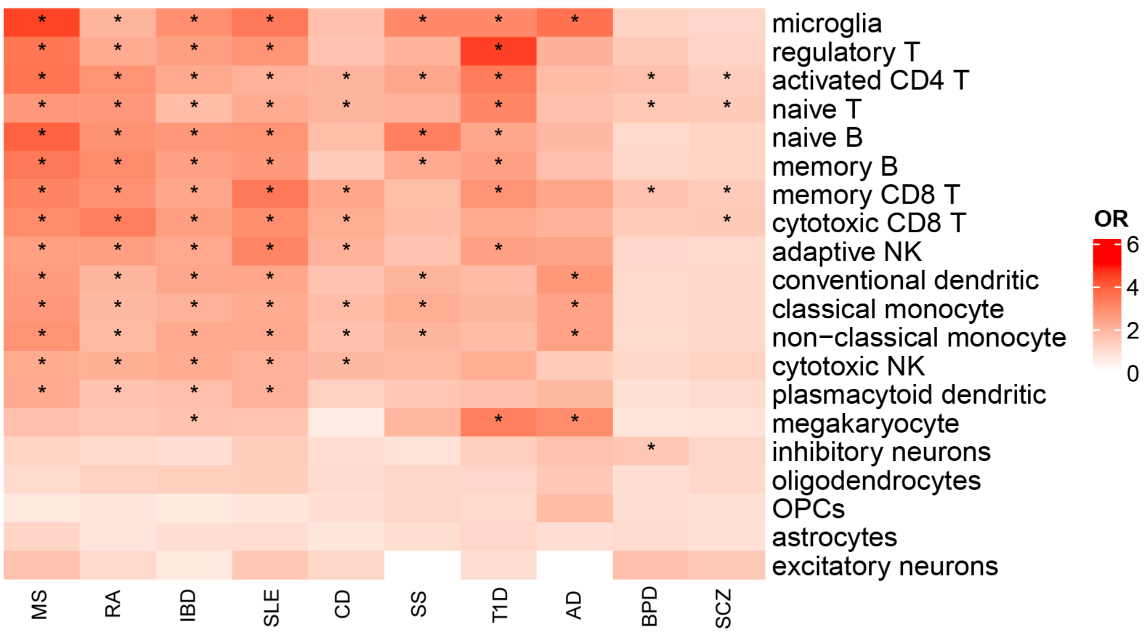

Supplementary Fig. 3. The heritability enrichment at cell-type-specific OCRs for GWAS associations of several immune-related and psychiatric disorders. In the heatmap, each column represents the odds ratio (OR) values for each disease and each row represents the OR values for each cell type. The color key from white to red represents the OR from low to high. The significant enrichments were marked with asterisks. MS, multiple sclerosis; RA, rheumatoid arthritis; IBD, inflammatory bowel disease; SLE, systemic lupus erythematosus; CD, celiac disease; SS, systemic sclerosis; T1D, type 1 diabetes; AD, Alzheimer disease; BPD, bipolar disorder; SCZ, schizophrenia.

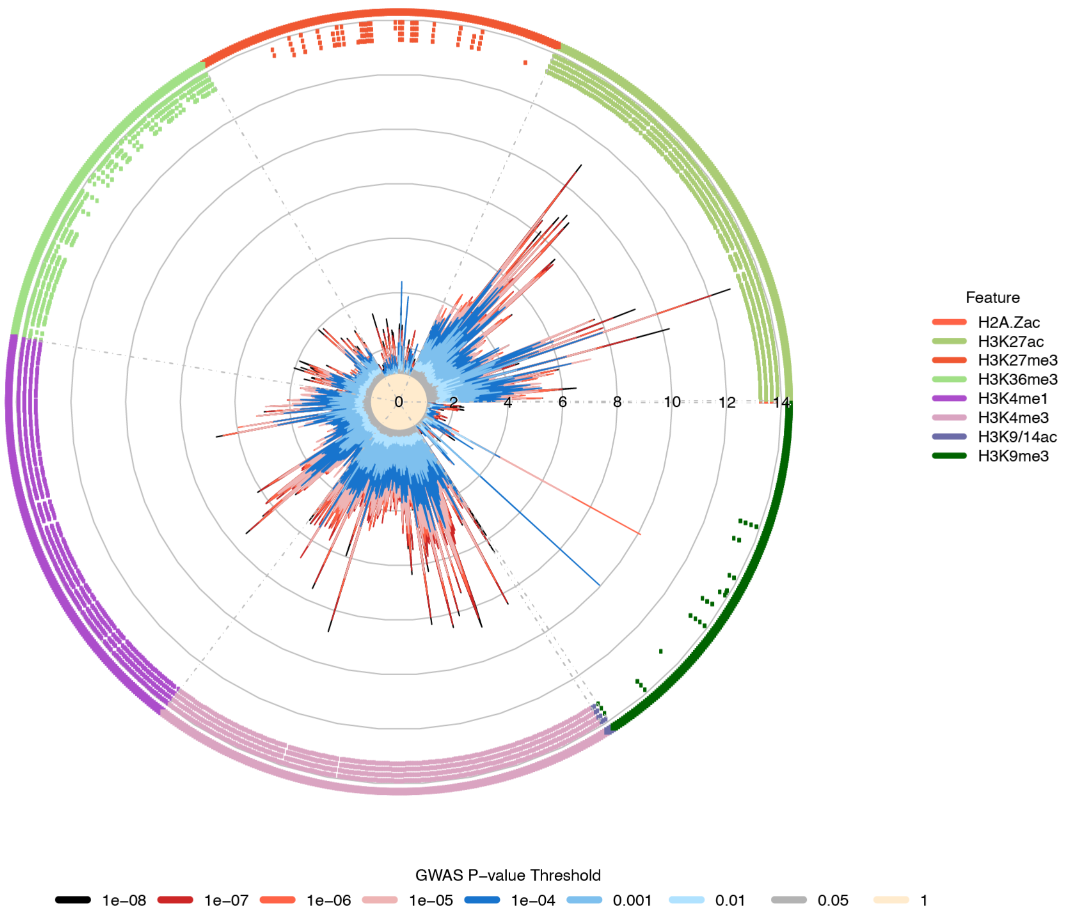

Supplementary Fig. 4. Enrichment of MS GWAS associations in histone modification ChIP-seq peaks. Radial lines show odds ratio (OR) values at eight GWAS P-value thresholds (T) for histone modification peaks of immune cell types available from the Blueprint project. Dots in the inner ring of the outer circle denote significant GARFIELD enrichment (if present) at T < 10−5 (outermost) to T < 10−8 (innermost) after multiple-testing correction. The colors represent different kinds of histone modifications.

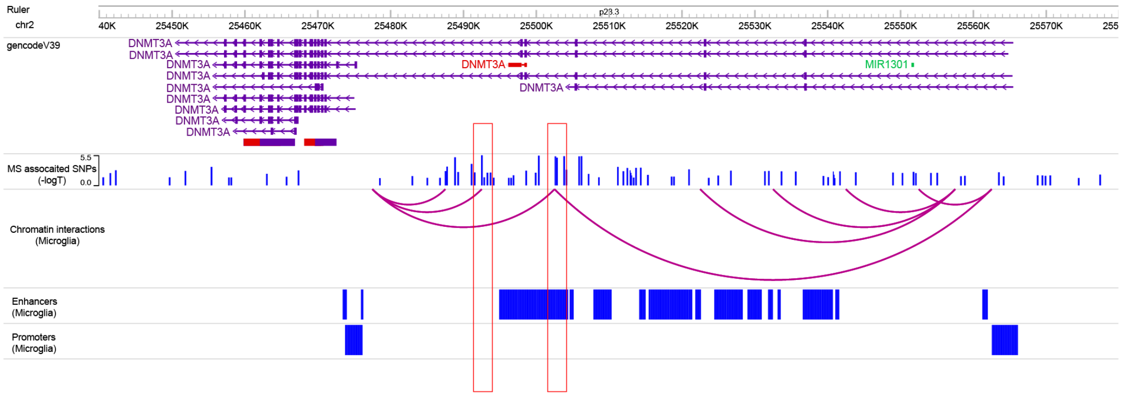

Supplementary Fig. 5. Visualization of DNMT3A locus on the reference human genome (hg19). Enhancer-promoter interactions, enhancer, and promoter regions in microglia are presented. Enhancer-promoter interactions are obtained from H3K4me3 HiChIP data. Enhancers and promoters are obtained from H3K27ac and H3K4me3 ChIP-seq data. Variants associated with MS susceptibility with nominal significance (T<0.05) in the MS GWAS are shown in the top. SNPs which are associated with MS susceptibility at T < 10−5 and interacted with promoter of *DNMT3A* gene are depicted with red boxes.

Supplementary Table 1. Prediction accuracy of CPRS in validation and test sets

Model (r^2 = 0.1)	**UKBB1** 601 cases/ 109,990 controls			**UKBB2** 1354 cases/ 252,065 controls			**UCSF-EPIC** 494 cases/ 449 controls		
	R^{2*} (%)	*P*	AUC (%)	R^{2*} (%)	*P*	AUC (%)	R^{2*} (%)	*P*	AUC (%)
**B cell**	2.2	4e-36	64.2	3.3	7e-119	66.8	4.2	2e-20	70
**Monocyte**	2.1	9e-35	64	3.2	3e-116	66	4.5	2e-21	70.7
**Microglia**	2.1	7e-35	63.8	3.1	1e-114	66.5	4.3	9e-21	70.5
**Combined**	2.1	3.9e-35	64	3.3	6.2e-120	67	4.5	2.9e-22	70

*Adjusted for MS prevalence 0.00127

Supplementary Table 2. Prediction accuracy of CPRS based on non-MHC variants in validation and test sets

Model (r^2 = 0.1)	**UKBB1** *601 cases/ 109,990 controls*			**UKBB2** *1354 cases/ 252,065 controls*			**UCSF-EPIC** *494 cases/ 449 controls*		
	R^{2*} (%)	*P*	AUC (%)	R^{2*} (%)	*P*	AUC (%)	R^{2*} (%)	*P*	AUC (%)
**B cell**	1.2	4e-19	59.9	1.9	5e-64	63	3.5	7e-18	66.7
**Monocyte**	1.1	3e-17	59.7	1.7	7e-56	62	3.8	5e-19	67
**Microglia**	1.5	1e-22	60.6	1.7	3e-55	61.8	3.7	1e-18	67
**Combined**	1.8	2e-28	60	2.1	3.4-69	63	4.3	4.7e-21	69

*Adjusted for MS prevalence of 0.00127

Supplementary Table 3. List of pruned Unique SNPs in each Cell Type

Cell Type	Unique SNP Sets
B cell	rs10411936, rs1051794, rs1059612, rs10793048, rs10877011, rs10905818, rs11023182, rs11079784, rs11129295, rs11164855, rs11587512, rs11625403, rs11755622, rs12179536, rs12198399, rs12210947, rs1233384, rs12365699, rs1250538, rs12603276, rs12713430, rs12772554, rs1321859, rs1383265, rs1441172, rs1445001, rs16957895, rs17189441, rs17206868, rs17291131, rs17668731, rs17797780, rs180524, rs2104286, rs2243148, rs2269426, rs2290324, rs233100, rs241432, rs248919, rs2636743, rs2844702, rs2875734, rs310224, rs3115627, rs3130171, rs3134930, rs3198502, rs34266569, rs354042, rs35998847, rs3763313, rs396090, rs4262739, rs427037, rs4288340, rs4403119, rs4648356, rs4821568, rs495089, rs4959041, rs4968318, rs547154, rs650258, rs653536, rs6589706, rs6705330, rs6753957, rs6795182, rs6906128, rs6962836, rs6965571, rs7087591, rs7123220, rs7174, rs7200786, rs7289534, rs746429, rs7679673, rs8107548, rs842283, rs902780, rs983494
Microglia	rs10029162, rs10158486, rs10469324, rs10478421, rs10512734, rs10786052, rs10797440, rs10905806, rs10947096, rs11582702, rs11666263, rs11808092, rs12212092, rs1250544, rs12623828, rs12643660, rs12716974, rs12897371, rs12945597, rs12946510, rs13106574, rs13190937, rs13192841, rs13195441, rs13255292, rs13386455, rs169494, rs169858, rs1704186, rs17119, rs17127169, rs175702, rs2006893, rs2045767, rs2205829, rs2239765, rs2248372, rs2395030, rs2400899, rs2517415, rs2517855, rs2517938, rs2534826, rs258816, rs2600671, rs2648839, rs28366155, rs2844651, rs2844670, rs2857281, rs28636834, rs28895018, rs2984920, rs3129727, rs3130048, rs3130279, rs3130980, rs3132587, rs34498363, rs35954087, rs3745619, rs3817171, rs3819250, rs384247, rs3892710, rs3893259, rs391755, rs401775, rs4077669, rs4147359, rs4394275, rs446853, rs4483030, rs453779, rs4613763, rs4713270, rs4742716, rs4796224, rs4821560, rs4902647, rs6454582, rs6533183, rs6671268, rs6788635, rs7193268, rs7256818, rs7275, rs731707, rs7453967, rs748048, rs7535068, rs7554511, rs7569084, rs7632991, rs7751505, rs8083368, rs816411, rs8888, rs915894, rs9262651, rs9263726, rs9263875, rs9267404, rs9391846, rs9625520, rs9877650
Monocyte	rs10056247, rs1015166, rs10484436, rs10493763, rs1063478, rs11000801, rs11130110, rs11187025, rs11757306, rs1177279, rs1264344, rs1264702, rs12883250, rs13111352, rs1366302, rs1367727, rs1432295, rs17035311, rs17051321, rs17124112, rs17470892, rs17642703, rs1881995, rs1984021, rs202568, rs2071477, rs2071541, rs2074473, rs2197025, rs2284190, rs2395300, rs2621323, rs2726455, rs2735097, rs2736177, rs2844472, rs2856830, rs3113494, rs3129304, rs3131064, rs3132935, rs377743, rs3819720, rs3930531, rs411326, rs4148876, rs4248166, rs4418214, rs447088, rs4821565, rs4902652, rs497239, rs547812, rs6019420, rs6451493, rs6906021, rs694739, rs7513382, rs769177, rs7767167, rs7774452, rs7796492, rs842252, rs867436, rs9262155, rs9276399, rs9295837, rs9380199

Supplementary Table 4. Phenotype Association of CPRS in EPIC

Phenotype	Combined		B cell		Monocyte		Microglia	
	R^2	β	R^2	β	R^2	β	R^2	β
**BV**	1.03	-0.08	1.07	-0.09	1.42	-0.10	0.98	-0.08
**WMV**	1.83*	-0.12	1.76*	-0.12	2.14*	-0.13	1.67*	-0.12
**pGMV**	0.04	-0.02	0.1	-0.02	0.15	-0.03	0.05	-0.02
**CSF**	0.78	0.08	0.59	0.09	0.88	0.08	0.91	0.08

**P* < 0.05
***P* < 0.01

Supplementary Table 5. Phenotype association of CPRS based on non-MHC variants in EPIC

Phenotype	Combined		B cell		Monocyte		Microglia	
	R^2	β	R^2	β	R^2	β	R^2	β
**BV**	1.81*	-0.11	0.96	-0.08	1.66*	-0.10	0.7	-0.07
**WMV**	1.98*	-0.13	0.94	-0.09	2.2*	-0.14	1.23	-0.10
**GMV**	0.53	-0.05	0.11	-0.02	0.22	-0.03	0.01	-0.01
**CSF**	0.91	0.08	0.63	0.07	0.55	0.07	0.25	0.04

**P* < 0.05
***P* < 0.01

Supplementary Table 6. Phenotype association of CPRS based on unique SNPs in UK Biobank

Phenotype	Combined		B cell		Monocyte		Microglia	
	R^2	β	R^2	β	R^2	β	R^2	β
**BV**	6.8	-0.27	10.2*	-0.47	2.9	-0.10	5.5	-0.21
**WMV**	5.8	-0.27	6.3	-0.40	3.2	-0.12	4.8	-0.21
**GMV**	2.2	-0.14	5*	-0.31	0.6	-0.44	1.7	-0.11
**CSF**	1.8	0.15	2.2	0.23	0.6	0.5	1.9	0.13

**P* < 0.05
***P* < 0.01

Supplementary Table 7. Phenotype association of CPRS of non-MS subjects in UK Biobank

Phenotype	Combined		B cell		Monocyte		Microglia	
	R^2 (%)	β	R^2 (%)	β	R^2 (%)	β	R^2 (%)	β
**BV**	3.55	1	4.11	0.92	2.19	0.99	0	0.21
**WMV**	4.11	1.6	4.77	1.97	2.54	1.06	0	0.8
**GMV**	3.35	0.19	3.87	-0.24	2.06	0.62	3	-0.34
**CSF**	0.1	2.82	0.1	3.71	2.38	0.2	0.1	3.51
