# Supplementary material for: Integration of epigenetic and genetic profiles identifies multiple sclerosis disease-critical cell types and genes
Source: Commun Biol. 2023 Mar 30;6:342. doi: 10.1038/s42003-023-04713-5 (PMC10063586; doi:10.1038/s42003-023-04713-5)
Supplement: Supplementary file 3 — Description of Additional Supplementary Files [file 42003_2023_4713_MOESM3_ESM.pdf]

Description of Additional Supplementary Files

File name: Supplementary Data 1

Description: The putative causal genes identified by H-MAGMA..

File name: Supplementary Data 2

Description: The common and unique risk genes.

File name: Supplementary Data 3

Description: The cell-specific risk genes overlapped with previously prioritized genes.

File name: Supplementary Data 4

Description: Gene ontology analysis on the common genes. The source data behind the figure 3b in the paper.

File name: Supplementary Data 5

Description: Gene ontology analysis on the unique genes. The source data behind the figure 3c in the paper.

File name: Supplementary Data 6

Description: List of pruned overlapping SNPs across cell-types.

File name: Supplementary Data 7

Description: The source data behind the figure 4 in the paper.

File name: Supplementary Data 8

Description: The source studies for the summary statistics.

File name: Supplementary Data 9

Description: Links for publicly available epigenetic datasets used in the study.
